# Marketing depression care management to employers: design of a randomized controlled trial

**DOI:** 10.1186/1748-5908-5-22

**Published:** 2010-03-16

**Authors:** Kathryn M Rost, Donna Marshall

**Affiliations:** 1 Department of Medical Humanities and Social Sciences, Florida State University College of Medicine, Tallahassee, Florida, USA; 2 Colorado Business Group on Health, Denver, Colorado, USA

## Abstract

**Background:**

Randomized trials demonstrate that depression care management can improve clinical and work outcomes sufficiently for selected employers to realize a return on investment. Employers can now purchase depression products that provide depression care management, defined as employee screening, education, monitoring, and clinician feedback for all depressed employees. We developed an intervention to encourage employers to purchase a depression product that offers the type, intensity, and duration of care management shown to improve clinical and work outcomes.

**Methods:**

In a randomized controlled trial conducted with 360 employers of 30 regional business coalitions, the research team proposes to compare the impact of a value-based marketing intervention to usual-care marketing on employer purchase of depression products. The study will also identify mediators and organizational-level moderators of intervention impact. Employers randomized to the value-based condition receive a presentation encouraging them to purchase depression products scientifically shown to benefit the employee and the employer. Employers randomized to the usual-care condition receive a presentation encouraging them to monitor and improve quality indicators for outpatient depression treatment. Because previous research demonstrates that the usual-care intervention will have little to no impact on employer purchasing, depression product purchasing rates in the usual-care condition capture vendor efforts to market depression products to employers in both conditions while the value-based intervention is being conducted. Employers in both conditions are also provided free technical assistance to undertake the actions each presentation encourages. The research team will use intent-to-treat models of all available data to evaluate intervention impact on the purchase of depression products using a cumulative incidence analysis of 12- and 24-month data.

**Discussion:**

By addressing the 'value to whom?' question, the study advances knowledge about one of the most pivotal problems in the translation of evidence-based care to 'real world' settings: whether purchasers can be influenced to buy healthcare products on the basis of value and not exclusively on the basis of cost. If value-based marketing increases depression product purchase rates over usual care, this study will provide encouragement to market new healthcare products on the basis of the product's value to the purchaser as well as the recipient of care.

**Trial Registration:**

Clinical Trials Registration Number: NCT01013220

## Background

Recent studies estimate that 7.6% of employees suffer a major depressive episode each year [1]. Depression substantially reduces an employee's ability to work, as evidenced by increased absenteeism [2-5] and reduced productivity at work (hereafter referred to as productivity) [2-7], with annual work costs approaching $24 billion (Y2K$) [1]. As the most prevalent disorder of the five conditions that cause the greatest work loss in the American workforce [8,9], depression will soon become the leading cause of disability in industrialized countries [10].

Employers can reduce their depression-related work losses by ensuring their employees receive the type, intensity and duration of depression care management shown to improve clinical and work outcomes in effectiveness trials [11-14]. Employers, who finance health insurance coverage for an estimated 90% of non-elderly individuals with private health insurance [15], can purchase products that increase the probability that their depressed employees receive this evidence-based care. Interventions to increase product purchase need to increase employer motivation and capacity to purchase.

### Increasing employer motivation to purchase

In the studies to date, employers report substantial information deficits about the costs that organizations absorb when depressed employees fail to receive adequate treatment. Employers who receive this information report interest in reviewing the data that depression products achieve a return on investment. Even more encouragingly, employers note that they are willing to apply program savings from improved absenteeism and productivity against program costs [16-24].

### Increasing employer capacity to purchase

Employers interested in purchasing a depression product that provides value face non-trivial challenges. Employers who contract with multiple health plans have to contract with an additional vendor (*e.g*., a disease management company or managed behavioral health organization) to provide a depression product to their workforce. Because the marketplace does not currently provide a list of vendors who sell depression products, interested employers often know only those products recommended by their colleagues. Not surprisingly, products differ substantially in their cost and capacity to deliver evidence-based services, requiring employers to make informed choices despite imperfect information to realize value for themselves or their employees. To address this need, this study provides technical assistance to employers to identify high-quality depression products, referring to products that provide the type, intensity, and duration of depression care management shown to improve clinical and work outcomes as Depression Management in the Workplace (DMW) products.

### Scope of Study

The specific aims of the study are: to compare the impact of value-based (VB) and usual-care (UC) intervention on employer purchase of depression products; to identify mediators of intervention impact on employer purchase; and to identify organizational-level moderators of employer purchase.

The first specific aim utilizes an experimental design to study intervention effectiveness. Hypothesis one tests whether VB intervention significantly increases purchasing behavior over UC. The second specific aim utilizes a non-experimental design to study intervention mediators. Hypothesis two tests whether intervention impact on purchasing behavior is mediated by the organization's appraisal of product benefit to the employer more than the employee. Hypothesis three tests whether colleagues influence an organization's appraisal of product benefit to employer. The third specific aim utilizes a non-experimental design to study intervention moderators. Hypothesis four tests whether larger and more mature companies with greater financial latitude demonstrate higher levels of purchasing behavior, as well as companies who make greater investments in their employees and have a higher tolerance for benefit risk, independent of intervention. Hypothesis five tests whether companies with de-centralized onsite purchasing groups in which the presentation participant has primary influence will demonstrate higher levels of purchasing behavior, independent of intervention. Hypothesis six tests whether companies with strong vendor relationships demonstrate higher levels of purchasing behavior, independent of intervention.

Before initiating the study, the research team: fully articulated a conceptual framework; pilot tested the VB intervention prototype to demonstrate intervention feasibility, to collect/integrate employer feedback to further strengthen the intervention, and to estimate effect size; created instrumentation to measure intervention mediators, moderators, and outcomes with demonstrated reliability and validity; investigated business coalition interest in participating in the study; and received approval from the Florida State University Institutional Review Board.

## Methods/design

### Participants and setting

#### Regional Coalitions

Employers join coalitions in their geographic area to identify innovative solutions to provide quality healthcare at affordable prices, focusing on benefit products for their non-unionized employees. The 58 coalition members of the National Business Coalition on Health (NBCH) are eligible to participate in the study if: they have 30 or more current employer purchasers as members/affiliates; have hosted presentations in regularly scheduled meetings during the past year (eliminating a limited number of coalitions who served exclusively as purchasing agents); and have not participated in the research team's preliminary studies. The research team, in conjunction with the NBCH Board of Directors, sends eligible coalitions an invitation to participate, followed up by a telephone call, describing the purpose of the study as testing two educational presentations on assuring high quality depression care.

#### Employers

Employers who belong to regional coalitions are eligible to participate if: they represent a public or private company that provides health benefits to 100 or more domestic employees; their company intends to remain in the regional coalition for the next two years; and the coalition's Executive Director does not indicate they have purchased depression products for all their employees in the past two years. Employers who join regional coalitions appoint one employee from their company to represent them. Unpublished studies indicate that more than 60% of these representatives report strong influence in benefit purchasing decisions. The Executive Director of each participating coalition distributes a fact sheet to all eligible representatives inviting them to participate in a study that tests two educational presentations about how companies can improve the depression treatment their employees receive. The Executive Director follows up with each member by telephone to confirm that 6 or more employers agree to participate in the study without knowing which condition they will be assigned to.

#### Randomization

**Figure 1:**
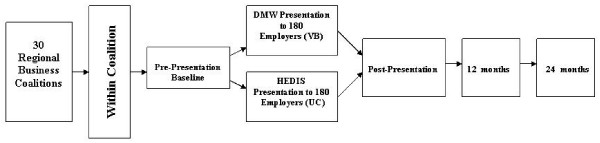
**Research Design**.

Participating coalitions are randomized to one of six quarters ending March 2011 for presentation to reduce historical threats to validity in non-experimental analyses. As shown in Figure 1, participating employers within a coalition are block randomized by workforce size to the VB or UC condition. After being alphabetized, all participating employers are assigned a unique two-digit number from a random numbers table created by the principal investigator at a centralized location blinded to all company names. Each participating employer is matched to another participating employer in the same coalition by workforce size before the employer with the higher number in each pair is randomized to the VB condition with the other member randomized to the UC condition. When randomization is completed in each coalition, a member of the research team works with the Executive Director to invite participating employers to the presentation to which they had been randomized. Participants remain blind to intervention condition until the presentation begins.

#### Intervention

The intervention consists of a presentation and technical assistance delivered to employer representatives at local meetings sponsored by regional coalitions. Employers randomized to the VB condition receive the Depression Management in the Workplace (DMW) presentation. Employers randomized to the UC condition receive the Healthcare Effectiveness Data and Information Set (HEDIS) presentation. All interested employer representatives are offered condition-specific technical assistance free of charge during the 24 months after the presentation.

#### Presentations

The DMW and HEDIS presentations present the content summarized in Table 1 utilizing high quality graphic material recently awarded The Communicators Award of Excellence in an international competition.

**Table 1:** Presentation Schematic

Sequence of Initial Activities	VB Presentation	UC Presentation
PRESENTATION	Prevalence in the workplace	Prevalence in society

	Depression burden to	
	Employer	Depression burden to individual
	Employee	

	Problems treating depression in usual care	Problems treating depression in usual care

	DMW as an indicator of high quality care	HEDIS as an indicator of high quality care

	Clinical effectiveness of DMW Care Organizational effectiveness of DMW	Obtaining HEDIS indicators for outpatient depression management

	DMW Calculator	Interpreting HEDIS indicators for outpatient depression management

	Description of Technical Assistance	Description of Technical Assistance

DISCUSSION	Open discussion of value of DMW Care	Open discussion of value of HEDIS quality care

#### DMW presentation

The two-hour DMW presentation educates employer representatives about DMW Care and its evidence-based impact on clinical and work outcomes. Employer representatives receive a company-specific return on investment (ROI) estimate associated with DMW Care. As shown in Table 2, this estimate is generated by a calculator the research team developed in its earlier studies by translating scientifically derived estimates of DMW Care's impact on absenteeism and productivity at work to a monetized savings in lost work days, varying pertinent employee, organizational, and vendor characteristics [25]. During the presentation, employers are encouraged to explore purchasing a depression product for their company and to request free technical assistance to help them purchase a DMW Care quality product.

**Table 2:** Calculator Schematic

Major Constructs	Definition
Size ^1^	Number of non-unionized domestic employees currently receiving health care benefits

Industry ^1,3^	Industry type allows calculator to estimate age by gender employee distributions to calculate depression prevalence

Hourly wage/fringe ^1,3,4^	Hourly wage plus BLS-estimated fringe for non-supervisory personnel in industry type

Missed work policies^1^	Paid sick leave policies
	Temporary employee policies
	Work makeup policies and practices

Depression in workforce^2,3^	Number of employees in workforce with 1-year major depression and/or dysthymia

Lost work days associated with Depression^2,3^	Workdays an employer pays for where work is never completed by temporary, coworkers or depressed worker when s/he feels better

DMW employee participation rate ^2,3,4^	Number of depressed employees expected to participate in DMW each year

DMW cost per employee participant^2,3,4^	Estimate cost per employee participant

Annual DMW impact on lost work days ^2,3^	Incremental reduction in lost work days in workforce using DMW employee participation estimate

Other potential payers ^2,3^	Summary of peer-reviewed literature on economic impact on health plans and employees

Performance standards ^2,3^	DMW key component operationalization

Annual DMW cost ^2,3^	Based on estimated participation rate and cost per employee participant

Annual DMW cost per reduced lost work day ("ROI") ^2,3^	DMW cost/incremental reduction in lost work days

^1^indicates user provides information.

^2^indicates calculator provides information.

^3^indicates calculator provides documentation/detailed description of estimate derivation and graph.

^4^indicates user can modify default values.

#### HEDIS presentation

The two-hour HEDIS presentation educates employers about HEDIS indicators for antidepressant medication management and their use in monitoring outpatient depression treatment quality. Employers receive HEDIS indicators for antidepressant medication management for their most subscribed plan if that plan reports its HEDIS scores to the National Committee for Quality Assurance; otherwise, they receive the HEDIS indicators for other plans in the area. During the presentation, employers are asked to encourage their most subscribed health plan to improve its HEDIS indicators for depression (or to calculate its HEDIS indicators if it does not report them). In addition, employers are encouraged to ask their plans to provide individual feedback to clinicians about the quality of their depression care, provide greater formulary access to newer depression drugs, and require lower copayments for outpatient mental healthcare. While causal evidence is lacking, a study reports that these plan characteristics are associated with better HEDIS indicators for antidepressant medication management [26]. Because previous studies indicate that the HEDIS presentation will have little to no impact on employer purchasing [27-31], depression product purchasing rates in the UC condition capture vendor efforts to market depression products to employers in both conditions during follow-up.

The second author (DM) provides presentations to both groups. DMW and HEDIS presentation sessions are scheduled for the same day in random order, one in the morning and the other in the afternoon. If after agreeing to be in the study, employer representatives fail to attend the meeting, they are asked to schedule a time in the next four weeks to complete the presentation and data collection individually. If they cannot do so, they are dropped from the study.

#### Technical assistance (TA)

TA is the provision of individualized consultation to enable employers to improve the depression care their employees receive. When an employer representative requests TA, the TA consultant schedules a two-hour phone call to conduct the initial consultation followed by a second call approximately one month later. In the VB condition, the TA assists employer representatives in building broad support within their organization for the purchase, in identifying DMW vendors, and in developing contracts for the program. In the UC condition, the TA consultant assists employer representatives to work with their most subscribed health plan to improve the depression treatment they deliver as measured by their outpatient antidepressant management HEDIS indicators, and/or to provide individual feedback to clinicians about the quality of their depression care, provide greater formulary access to newer depression drugs, and require lower copayments for outpatient mental healthcare.

#### Data collection

All employer representatives are asked to complete the pre-presentation survey immediately before the presentation begins, the post-presentation survey immediately after the presentation ends, as well as a 12- and 24-month follow-up survey. Twenty-four-month follow-up surveys are projected to be completed by September 2013. Employers are paid $100 for completing the pre- and post-presentation survey, $100 for completing the 12-month survey, $100 for completing the 24-month survey, and an additional $50 for completing all surveys. Pre- and post-presentation data are collected in the room in which the presentations are delivered using laptop computers. Twelve- and 24-month follow-up data are collected in the subject's office or home using the web. The research team member who actively contacts employers who do not respond to a standardized electronic cue to complete follow-ups is blinded to condition. Pre-presentation data (descriptive characteristics, mediating, moderating, and outcome variables) are collected from employer representatives immediately before the presentation. Post-presentation data (mediating variables and presentation evaluation) are collected from employer representatives immediately after the presentation. Twelve and 24-month data (mediating, selected moderating and outcome variables) are collected in a three month window of the expected timeframe. Employers whose representatives are no longer in the position or with the company are asked to nominate another representative to complete the presentation and remaining follow-up interviews.

The research team also conducts semi-structured interviews with Executive Directors of each participating coalition at baseline (two weeks before the presentation) and at 24-month follow-up. Executive Director baseline interviews provide qualitative data about coalition efforts to encourage VB purchasing. Executive Director follow-up interviews are designed to provide qualitative data on intervention impacts that may not be observable in the structured interviews we conducted with employers, as well as solicit insights from Executive Directors about VB intervention impact and strengthening. Instrumentation is available on the project's website [32].

#### Construct Measurement

Employer benefit purchasing behavior (EBPB) over the previous 12 months will be measured at 12 and 24 months as an ordinal variable with four levels: product exposure (*e.g*., presentation participation) only; product exposure and discussion with decision-maker only; product exposure, discussion with decision-maker and product pursuit; and product exposure, discussion with decision making, product pursuit, and product purchase. Planned secondary analyses will examine intervention impact on product purchase defined as a dichotomous variable. Descriptive, moderating, and mediating variables will be defined in subsequent manuscripts testing the study's hypotheses.

#### Data Analysis

The research team will test the experimental hypothesis using an intent-to-treat model of all available data, conducting a cumulative incidence analysis over 24 months. Assuming 20% dropout at 24 months (remaining n = 144/group), the post-attrition sample will provide 86% power to find a 0.35 effect size on the EBPB scale using a two-tailed test with p < 0.05.

## Discussion

Depression products have potential to reduce the toll depression exacts on employers by increasing the delivery of evidence-based care. This trial will determine if an intervention that emphasizes value to the healthcare purchaser as well as to the healthcare recipient can increase product purchase. By addressing the 'value to whom?' question, the study advances knowledge about one of the most pivotal problems in the translation of evidence-based care to 'real world' settings: whether purchasers can be influenced to buy healthcare products on the basis of value rather than only on the basis of cost. In the likely event that VB > UC, the study will provide encouragement to market evidence-based healthcare to purchasers on the basis of the value the organization itself will realize. UC may achieve comparable outcomes to VB if the limiting factors in benefit purchasing are organizational, purchasing group and vendor constraints that no intervention can meaningfully modify. Support for this scenario would encourage the targeted marketing of evidence-based healthcare to purchasers with empirically identified organizational, purchasing group, and vendor characteristics, using usual care strategies.

## Competing interests

The authors declare that they have no competing financial or non-financial interests. KR developed, directed, and published the intervention study used in part to define DMW Care.

## Authors' contributions

KR conceived of and designed the study, developed the instrumentation, and drafted the manuscript with assistance from the technical writer. DM made substantial contributions to the study questions to increase the interest of the study to employers; made suggestions to increase the feasibility of intervention implementation and data collection; supervises data collection, and revised the intellectual content of the manuscript. Both KR and DM have read and given final approval of the version to be published, and participated sufficiently in the work to take public responsibility for the content.
